# Coupled and Simultaneous Thermal Analysis Techniques in the Study of Pharmaceuticals

**DOI:** 10.3390/pharmaceutics15061596

**Published:** 2023-05-25

**Authors:** Marek Wesolowski, Edyta Leyk

**Affiliations:** Department of Analytical Chemistry, Faculty of Pharmacy, Medical University of Gdansk, Gen. J. Hallera 107, 80-416 Gdansk, Poland; edyta.leyk@gumed.edu.pl

**Keywords:** coupled techniques, simultaneous techniques, pharmaceuticals, TG, TG–FTIR, TG–MS, TG–GC/MS, DSC, DSC–photovisual, DSC–FTIR microspectroscopy, DSC–XRD

## Abstract

Reliable interpretation of the changes occurring in the samples during their heating is ensured by using more than one measurement technique. This is related to the necessity of eliminating the uncertainty resulting from the interpretation of data obtained by two or more single techniques based on the study of several samples analyzed at different times. Accordingly, the purpose of this paper is to briefly characterize thermal analysis techniques coupled to non-thermal techniques, most often spectroscopic or chromatographic. The design of coupled thermogravimetry (TG) with Fourier transform infrared spectroscopy (FTIR), TG with mass spectrometry (MS) and TG with gas chromatography/mass spectrometry (GC/MS) systems and the principles of measurement are discussed. Using medicinal substances as examples, the key importance of coupled techniques in pharmaceutical technology is pointed out. They make it possible not only to know precisely the behavior of medicinal substances during heating and to identify volatile degradation products, but also to determine the mechanism of thermal decomposition. The data obtained make it possible to predict the behavior of medicinal substances during the manufacture of pharmaceutical preparations and determine their shelf life and storage conditions. Additionally, characterized are design solutions that support the interpretation of differential scanning calorimetry (DSC) curves based on observation of the samples during heating or based on simultaneous registration of FTIR spectra and X-ray diffractograms (XRD). This is important because DSC is an inherently non-specific technique. For this reason, individual phase transitions cannot be distinguished from each other based on DSC curves, and supporting techniques are required to interpret them correctly.

## 1. Introduction

Pharmaceuticals (drugs) is a very general term in pharmacy that includes not only pharmaceutical preparations, but also substances for pharmaceutical purposes. Pharmaceutical preparations (drug preparations, drug formulations) are defined as complete dosage forms containing prescribed doses of active pharmaceutical ingredients and intended for use in humans or veterinary medicine [1]. Pharmaceutical preparations include substances for pharmaceutical purposes, i.e., active pharmaceutical ingredients (APIs, medicinal substances, drug substances) [2] and excipients [3]. APIs are responsible for the pharmacological action of drug preparations, while excipients enable the manufacture of preparations with optimal dosage, stability and bioavailability [2,3]. The proper selection of excipients is extremely important, as they enable the preparation of drug formulations that can resist gastric fluids, prevent vomiting and nausea, reduce or mitigate the undesirable taste and odor associated with oral drug administration, ensure that a high concentration of APIs are achieved at the target site and enable the manufacture of drug forms with delayed or prolonged therapeutic effects [3]. Most drug preparations are available on the pharmaceutical market in solid dosage forms, such as classic tablets (coated or uncoated), extended-release (controlled-release) tablets, irritants, suppositories and capsules containing extended-release powders, granules or pellets.

The development and manufacture of drug formulations that are effective in action, safe to use and stable require specialized studies, mainly involving APIs [4]. In addition to the standardly evaluated parameters such as API solubility and dissolution behavior, ionization constant, partition coefficient and stability, the physicochemical properties of APIs in the solid state, including crystalline/polymorphic forms, amorphism, hydration/dehydration and mechanical properties must also be determined [5,6]. One of the most useful ways to evaluate the physicochemical properties of APIs in the solid state is thermal analysis methods [7,8]. They are particularly useful because of the small amount of sample needed for analysis (up to a few milligrams), simple sample preparation and rapid measurement process. Among thermal analysis methods, three are most commonly used in the study of solid pharmaceuticals: differential scanning calorimetry (DSC), differential thermal analysis (DTA) and thermogravimetry (TG).

The main areas of DSC application in the study of pharmaceuticals are the exploration of phase transitions with simultaneous changes in the physical state of the API (melting, evaporation, sublimation, crystallization, glass transition, polymorphic and amorphous-crystalline transitions) and the study of chemical reactions with simultaneous changes in the chemical structure of the API (hydration, dehydration, desolvation, degradation and thermal decomposition) [7,8]. From the point of view of drug formulation technology, very important areas of the application of DSC are to understand the effects of physical processes (mixing, grinding, crushing, kneading and granulation) and thermal processes (spray-drying, freeze-drying, hot-melt extrusion and tableting) on the properties of the API (crystalline transformations, stability and bioavailability). TG, on the other hand, is mainly used to study API degradation processes occurring by solvolysis, oxidation, photolysis or pyrolysis; the characteristics of heating behavior; solid and volatile degradation products; and the mechanism and kinetics of thermal decomposition.

It is currently recognized that more than one measurement technique is required to reliably interpret the phase transitions and chemical reactions occurring in the samples during their heating or cooling in a controlled way. This is due to the need to eliminate the uncertainty associated with comparing data obtained by two or more single techniques when interpreting results obtained from two different samples taken from the same material and explored at different times. Hence, to prevent erroneous evaluation of the data obtained, it is desirable to study the same sample simultaneously, at the same time, using two or more measurement techniques [9,10]. Accordingly, the purpose of this review paper is to briefly characterize thermal analysis techniques used simultaneously or coupled with non-thermal techniques. This group of analytical techniques is finding increasing application in the study of APIs, since the results obtained using thermal analysis are enriched by data obtained for the same sample, and most often at the same time, using non-thermal techniques. The most common of these are chromatographic and spectroscopic techniques, which provide information on the qualitative and quantitative composition as well as changes in the chemical and crystalline structure of the samples during heating under controlled conditions.

The results discussed in this review prove beyond any doubt that coupled TG systems and simultaneous DSC measurements can play a key role in the study of pharmaceuticals. The data presented here show that without the use of TG-coupled techniques, it is not possible to identify volatile degradation products and determine the mechanism of thermal degradation of drug substances. In turn, simultaneous techniques facilitate the interpretation of DSC curves, which is particularly important in the study of phase transitions of drug substances. All this is important given that both thermal techniques (TG and DSC) are non-specific, and their interpretation often leads to ambiguous results. For these reasons, an additional goal of the review is to show the high utility of coupled and simultaneous systems in pharmaceutical technology.

## 2. Coupled and Simultaneous Techniques

Today’s thermal analysis equipment provides ample opportunities for the combined use of TG and DSC techniques with other techniques. Currently, three groups of combined measurement techniques are used in the study of pharmaceuticals, which are defined as simultaneous techniques, coupled simultaneous techniques and non-continuous simultaneous techniques (discontinuous simultaneous techniques) [11,12].

According to the International Confederation for Thermal Analysis (ICTA) recommendation on nomenclature in thermal analysis [11], the term “simultaneous techniques” is defined as “the application of two or more techniques to the same sample at the same time”, e.g., simultaneous DTA and TG analysis. The term “coupled simultaneous techniques” includes “the application of two or more techniques to the same sample when the two instruments involved are connected through an interface”, e.g., coupling a TG device with an FTIR spectrometer. In contrast, the term “discontinuous simultaneous techniques” includes “the application of coupled techniques to the same sample when sampling for the second technique is discontinuous”. An example is the coupling of a TG device with a gas chromatograph and mass spectrometer (GC/MS). In this case, appropriate portions of volatile degradation products are taken from the sample placed in the TG furnace and successively subjected to GC/MS analysis.

### 2.1. Thermogravimetry (TG)

**Figure 1:**
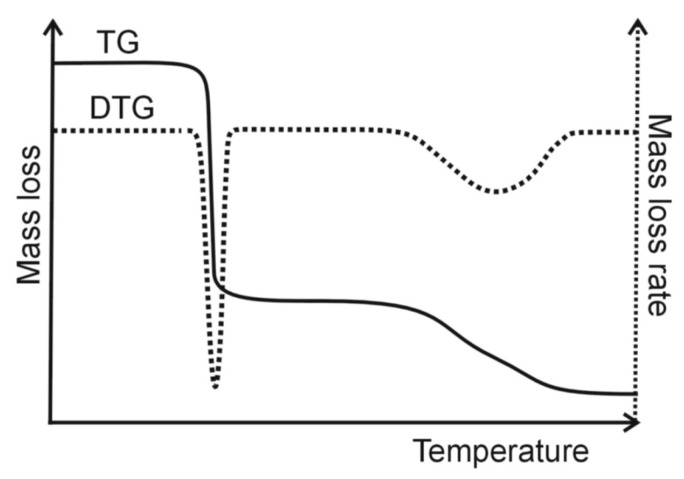
Typical thermogravimetric (TG) and differential thermogravimetric (DTG) curves.

As previously mentioned, TG is one of the most widely used thermal analysis methods in the study of pharmaceuticals. In TG, the change in a sample mass (∆m, loss or gain) is measured as a function of temperature, whilst the sample is subjected to a controlled temperature program [13]. Changes in mass are recorded as a function of temperature, obtaining a TG curve (Figure 1). TG instruments also allow simultaneous recording of the first derivative of the TG curve with respect to temperature, i.e., the DTG (Differential Thermogravimetry) curve. It illustrates the rate of change of sample mass as a function of temperature (dm/dT). As shown in Figure 1, when there are no changes in the sample related to mass loss or gain, the TG and DTG curves run parallel to the temperature axis. In turn, the deviation of the TG and DTG curves from the baseline illustrates the change in sample mass (TG curve) and the rate of mass change (DTG curve). The TG method is particularly useful for studying those physical transformations and chemical reactions that are accompanied by a change in mass.

Since TG is a non-specific technique, i.e., it does not provide direct data on the nature of the mass changes taking place, TG devices are widespread as coupled thermal techniques. The purpose of researching TG-coupled techniques is to analyze the evolved gas (Evolved Gas Analysis, EGA). The following techniques are of greatest importance in this research: TG–FTIR—coupling the TG device to an FTIR spectrometer, TG–MS—coupling the TG device to a mass spectrometer (MS) and TG–GC/MS—coupling the TG device to a GC equipment with MS detection [14,15,16,17].

In the first two techniques (TG–FTIR and TG–MS), measurements take place in real-time, i.e., at the moment when volatile degradation products are released from the sample while it is being heated under controlled conditions, i.e., at a certain heating rate and using a certain type of gas atmosphere. In this method, TG curves and FTIR or MS spectra of the samples, which are contained in TG containers (crucibles), are performed at the same temperature and at the same time, as in the case of measurements taking place with the conventional TG technique. The third technique, TG–GC/MS, is characterized by the fact that measurements do not take place in real time. Volatile degradation products that are released from the sample during heating are properly collected over specific temperature ranges. For example, they can be adsorbed by special adsorbents, and then, after desorption, they are subjected to GC/MS analysis. The results obtained from the analysis of MS spectra can be assigned to specific mass losses or temperature ranges on the TG curve, i.e., those ranges where volatile degradation products were collected.

### 2.2. Differential Scanning Calorimetry (DSC)

Specialized scientific journals often use the terms “coupled” or “combined” when DSC is used in combination with an optical microscope or FTIR spectrometer [18,19]. However, these combinations do not meet either the definition of “coupled techniques” or “combined techniques”. The nomenclature recommended by the International Confederation for Thermal Analysis and Calorimetry (ICTAC) defines the term “combined techniques” as “the application of two or more techniques to the different samples at the same time” [12]. Consequently, measurements using a combination of DSC with other techniques are in fact “simultaneous”, that is, “the application of two or more techniques to the same sample at the same time”.

**Figure 2:**
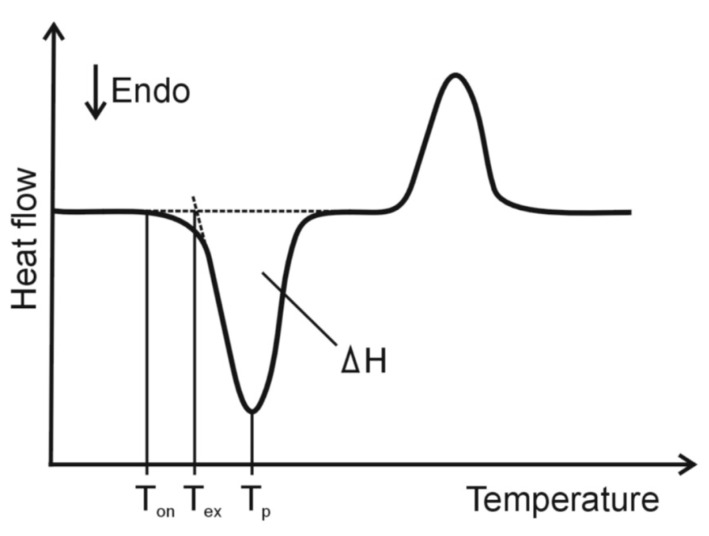
A typical differential scanning calorimetry (DSC) curve. T_on_—onset temperature of peak, T_ex_—extrapolated onset temperature of peak, T_p_—peak temperature and ΔH—heat of transition typical thermogravimetric (TG) and differential thermogravimetric (DTG) curves.

The DSC method is fundamentally different from TG. In DSC, the difference in energy inputs into a sample and a reference material (ΔH) are measured as a function of temperature whilst the sample and the reference material are subjected to a controlled temperature program [20,21]. As the reference material, an empty pan is the most frequently used. The heat exchanged by the sample with the environment is recorded as a function of temperature, yielding a DSC curve (Figure 2). Depending on the method of measurement, there are two types of DSC, power compensation DSC and heat flux DSC. According to the ICTAC definition [12], the power compensation DSC is a “technique where the difference between the electrical powers into a sample and a reference material is measured”, while the heat flux DSC is a “technique where the difference between the heat flow rates into a sample and a reference material is measured”. As shown in Figure 2, the DSC curve runs parallel to the temperature axis when the temperature of the sample and reference material have the same value, i.e., no heat transfer processes are taking place in the sample. If, on the other hand, the temperature of the sample is lower than that of the reference material, there is a process in the sample that requires the supply of external heat energy (the endothermic DSC peak). In the opposite case, if the temperature of the sample is higher than that of the reference material, there is a process in the sample involving the release of thermal energy (the exothermic DSC peak). DSC is particularly useful for studying physical transitions and chemical reactions accompanied by sufficient heat exchange with the environment. It is primarily a method of phase analysis.

The purpose of using DSC simultaneously with other measurement techniques stems from the fact that DSC is an inherently non-specific technique. For example, different phase transitions cannot be distinguished from each other based on a DSC curve, which can greatly complicate the interpretation of DSC data. In contrast, it is very easy to distinguish phase transitions using a microscope, which allows direct observation of phenomena occurring in the sample during heating or cooling under controlled conditions. This led to the introduction of new simultaneous techniques for the study of pharmaceuticals, in which the non-thermal technique was a microscope and/or digital camera (DSC–photovisual), FTIR microspectrometer (DSC–FTIR microspectroscopy) or XRD diffractometer (DSC–XRD).

All three simultaneous techniques make it possible to obtain information about the sample from a single experiment. Observation of the sample and recording of FTIR spectra or XRD diffractograms are performed at the same time and temperature, just like conventional DSC measurements. DSC–photovisual allows direct observation of thermal transformations occurring in the sample during heating or cooling under DSC conditions. DSC–FTIR provides information about the thermal and spectral properties of the sample. Thus, it is possible to monitor the changes occurring in the chemical and physical composition of the sample during heating or cooling. DSC–XRD, on the other hand, provides information on thermal properties and crystal structure. In this way, the crystalline-amorphous transformations of pharmaceuticals can be monitored.

## 3. Coupled TG Measurements

The coupling of TG devices with non-thermal techniques has proven remarkably useful. Data obtained using coupled TG systems with FTIR, MS or GC/MS are crucial from the viewpoint of drug formulation technology. They make it possible to know exactly how APIs behave during heating, identify volatile degradation products and determine the mechanism of thermal degradation. These data make it possible to predict the behavior of APIs during the manufacture of pharmaceutical formulations and enable the determination of their shelf life and storage conditions.

### 3.1. TG Coupled to FTIR Spectrometer

**Figure 3:**
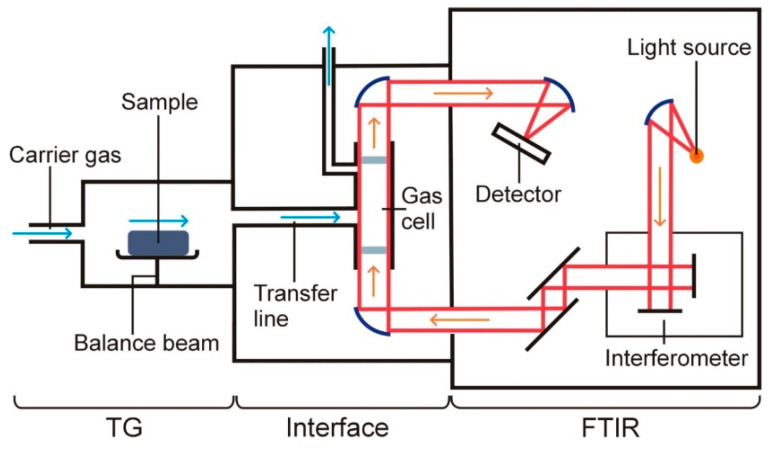
A block diagram of the equipment for TG–FTIR measurements.

Of all the techniques coupled to the TG device, TG–FTIR is the simplest and, at the same time, the most widely used coupled system in pharmaceutical studies. A block diagram of the TG–FTIR equipment is shown in Figure 3. In addition to the TG and FTIR devices, the most important element of this system is the interface, which connects the TG device to the FTIR spectrometer [14,15,16]. The interface includes the measurement chamber (gas cell) of the FTIR spectrometer and a connector (transfer line) that connects the outlet of the TG oven to the inlet of the measurement chamber of the FTIR spectrometer.

The measurement cell is installed in the light line between the interferometer and the detector. The measurement chamber and the connector are maintained at a certain temperature, which prevents condensation of volatile degradation products on the inner walls of these components. The most suitable temperature at which the measuring chamber and connector should be maintained is that at which no thermal degradation of the sample under study occurs. In turn, the carrier gas carrying the volatile degradation products from the TG oven to the measuring chamber of the FTIR spectrometer is the same gas in which the sample was explored in the TG furnace. It should be mentioned that both inert and oxidizing gas atmospheres can be used for TG–FTIR.

Literature data showed that coupled TG–FTIR systems have been widely used for studying APIs, while no example was found of the use of TG–FTIR for studying pharmaceutical preparations (tablets, capsules, irritants). It should also be mentioned that the equipment and measurement conditions used for the study varied widely, as shown in Table 1. Analysis of these data indicates that a device from TA Instruments, model SDT-Q600 TG/DTG/DTA, and an FTIR spectrometer from Thermo Scientific, model Nicolet iS10, were most often used to study APIs.

Volatile degradation products were obtained by decomposing the samples in a TG device. Various mass samples were used, usually 10 mg [22,23,24,25,26,27], 12–15 mg [28,29,30,31,32] or 15–20 mg [24,33]. Samples in open α-alumina sample holders [24,25,26,27,28,29,30,31,32] or ceramic crucibles [34] were heated from ambient temperature to 400 °C [26,27], 500 °C [34], 700 °C [35] or 800 °C [22,23]; at heating rates of 5 °C/min [34], 10 °C/min [28,29,30,31,32] or 20 °C/min [22,23,24,25,26,27,35]; in dry nitrogen [22,23,26,27,28,29,30,31,32,34,35] or dry air [22,23,24,25,35]; and at flow rates of 20 mL/min [34], 50 mL/min [24,25,28,29,30], 60 mL/min [31,32], 80 mL/min [35] or 100 mL/min [22,23,26,27].

The data summarized in Table 1 also indicate that the connector between both instruments, i.e., the TG device and the FTIR spectrometer, with a length of 120 cm and an inner diameter of 2–3 mm, was made of stainless steel. In only one case, the connector was made of Teflon. The connector and the measuring chamber of the FTIR spectrometer were mostly maintained in the temperature range of 200–270 °C. In only one case, both elements were not heated and were kept at 25 °C. On the other hand, FTIR spectra were recorded in the spectral range from 4000 cm^–1^ to 675–400 cm^–1^, most often with a resolution of 4 cm^–1^, using a Deuterated TriGlycine Sulfate (DTGS) detector. The interferometer and the gas cell compartment were purged with nitrogen [28,29].

**Table 1:** TG–FTIR coupled systems used in the study of active pharmaceutical ingredients.

TG Instruments	FTIR Spectrometers	Transfer Line	FTIR Spectra Measurements	References
SDT-Q600 TG/DTG/DTA (TA Instruments)	Nicolet iS10 FTIR (Thermo Scientific)	stainless steel tube, l = 120 cm, ø = 2 mm; 200 °C, 220 °C, 225 °C, 230 °C	200 °C, 220 °C, 250 °C; nitrogen, flow rate 50 mL/min; 4000–500 cm^–1^, 32 scans, 4 cm^–1^, 6 cm^–1^; DTGS (KBr)	[22,23,28,29,30,31,32,35,36]
TGA/DSC Stare (Mettler-Toledo)	Nicolet iS10 FTIR (Thermo Scientific)	stainless steel tube, l = 120 cm, ø = 3 mm; 25 °C, 200 °C	25 °C, 250 °C; air, flow rate 50 mL/min; 4000–600 cm^–1^, 32 scans, 4 cm^–1^; DTGS (KBr)	[24,25,37]
TGA/SDTA 851 (Mettler-Toledo)	Nicolet iS10 FTIR (Thermo Scientific)			[38]
TG-DSC 1 (Mettler-Toledo)	FTIR Nicolet (Thermo Scientific)	stainless steel tube, l = 120 cm, ø = 3 mm; 225 °C	250 °C; air, nitrogen, flow rate 50 mL/min; 4000–675 cm^–1^, 16 scans, 4 cm^–1^; DTGS (ZnSe, KBr)	[39,40,41,42]
SDT-Q600 TG/DTG/DTA (TA Instruments)	Nicolet 6700 FTIR (Thermo Fisher Scientific)		8 scans, 4 cm^–1^; MCT-A	[33]
TGA 2950 (TA Instruments)	Nexus 470 FTIR (Thermo/Nicolet)	250 °C	250 °C; air; 4000–450 cm^–1^, 32 scans, 4 cm^–1^	[43]
TG 2050 (TA Instruments)	FTS 3000 IR (BioRad Excalibur)	stainless steel tube	4 cm^–1^	[44]
STA 6000 TG (Perkin Elmer)	Frontier FTIR (Perkin Elmer)	270 °C	4000–450 cm^–1^	[34]
Diamond TG/DTG/DTA (Perkin Elmer)	Spectrum 100 (Perkin Elmer)			[45]
STA 449 Jupiter F1 TG/DTG/DSC (Netzsch)	FTIR TGA 585 (Bruker)	Teflon transfer line, ø = 2 mm; 200 °C	200 °C; 4000–600 cm^–1^, 16 scans, 4 cm^–1^	[46]
TG 209 (Netzsch)	IFS 66 (Bruker)			[47]
Setsys 16 TG-DTA/DSC (Setaram)	Thermo Nicolet Nexus 670 FTIR (Thermo Scientific)	stainless steel tube, l = 100 cm, ø = 3 mm, 200 °C	200 °C; 8 scans, 8 cm^–1^	[26,27]

DTGS—deuterated triglycine sulfate detector; l—length of transfer line; MCT-A—mercury cadmium telluride detector; ø—inner diameter of the transfer line.

Recently, a new instrument for TG–FTIR was introduced to the market, in which an apparatus for simultaneous TG–DSC measurements (STA 449 Jupiter, Netzsch) was directly coupled to an FTIR spectrometer (Bruker) without the use of a transfer line [48]. Mounting the FTIR spectrometer with very small dimensions directly on the top cover of the TG furnace eliminates the time delay in recording FTIR spectra due to the volume of volatile degradation products in the transfer line. The utility of this apparatus for evolved gas analysis was confirmed by studying the degradation of naphthalene, straw and topaz (a mineral).

Table 2 shows the general characteristics of APIs studying with coupled TG–FTIR systems. These data indicate that identifying volatile degradation products is one of the basic conditions for correctly determining the mechanism of thermal degradation. The utility of TG–FTIR in the study of APIs can be demonstrated by the example of naproxen ((+)-(*S*)-2-(6-methoxynaphthalen-2-yl)-propionic acid) and ketoprofen ((*RS*)-2-(3-benzoyl phenyl)-propionic acid) [29].

TG and DSC studies revealed differences in the thermal degradation of naproxen in nitrogen and air. In nitrogen, naproxen degrades with complete mass loss in one stage, in the temperature range of 154–305 °C, without forming any residue. In air, mass loss was found in two stages; the first stage was associated with 97% mass loss in the temperature range of 153–366 °C. The mass loss in the second stage was 1.5% and was due to afterburning of the coked residue. A sharp endothermic DSC peak in nitrogen and air confirmed the melting of naproxen at 153.5 °C. Subsequent endothermic and exothermic peaks reflected naproxen degradation, especially in the air. In turn, TG–FTIR analysis showed the presence of 2-methoxynaphthalene and propionic acid among the volatile degradation products. Characteristic FTIR bands indicating the presence of 2-methoxynaphthalene were found at 3100–2900 cm^–1^ and 1250–1000 cm^–1^, while bands characteristic of propionic acid were observed at 1780–1750 cm^–1^. Unlike naproxen, ketoprofen shows a complete mass loss in one step, in the temperature range of 164–329 °C in nitrogen and 156–338 °C in air. The sharp endothermic DSC peak was attributed to the melting of ketoprofen at 93.3 °C, while the second endothermic peak likely reflected evaporation. Evaporation of melted ketoprofen rather than decomposition was also confirmed by the absence of a coked residue (TG curve) and volatile degradation products (TG–FTIR).

Studies conducted showed that ketoprofen evaporates after melting without undergoing any thermal degradation. This was also confirmed using isothermal TG carried out at 210 °C in nitrogen. Under these conditions, both ketoprofen and naproxen evaporate from the melted sample without degradation. In contrast to ketoprofen, naproxen undergoes thermal degradation during dynamic heating, especially in the air. This was confirmed with the volatile degradation products and the coked-out decomposition residue.

Another example is the decomposition of oxytetracycline hydrochloride ((4*S*,4a*R*,5*S*,5a*R*,6*S*,12a*S*)-4-(dimethylamino)-3,5,6,10,11,12a-hexahydroxy-6-methyl-1,12-dioxo-1,4,4a,5,5a,6,12,12a-octahydrotetracene-2-carboxamide hydrochloride) [30]. TG measurements in the air indicated three stages of mass loss in the temperature ranges of 25–128 °C, 128–339 °C and 339–644 °C, respectively. This was confirmed by the DTA curve. The endothermic peak at 52 °C reflected the dehydration and release of hydrochloride during the first mass loss. A sharp exothermic DTA peak at 215 °C confirmed oxidative degradation in the second stage, while two exothermic peaks at 531 and 659 °C corresponded to the combustion of the coked residue in the last stage. TG–FTIR indicated that water and hydrochloride appear in the first stage of mass loss, while isocyanic acid, carbon dioxide, dimethylamine and ammonia were identified in addition to water and hydrochloride in the second stage. At approx. 510 °C, the final decomposition revealed the presence of methane. The TG, DTA and TG–FTIR studies enabled the development of an indicative mechanism of the thermal decomposition of oxytetracycline hydrochloride.

Studies have shown that, regardless of the identification of volatile degradation products, data on solid degradation products are also required to develop an accurate mechanism of the thermal degradation of APIs. Such a study was performed by determining the mechanism of thermal degradation of metoprolol tartrate (1-[4-(2-methoxy ethyl)-phenoxy]-3-(propan-2-ylamino)-propan-2-ol tartrate) [31]. TG and DTA showed that API degradation occurred with two or three mass losses depending on the atmosphere in which the degradation was carried out. TG in nitrogen indicated two consecutive mass losses, 22% in the 155–232 °C temperature range and 77% in the 232–653 °C range. The degradation processes were confirmed by two endothermic DTA peaks in these temperature ranges. In the air, similar results were obtained; the mass losses and temperature ranges for three consecutive stages were as follows: 22, 73 and 6% in the 153–229, 229–445 and 445–1000 °C ranges, respectively. The degradation processes were confirmed by endo- and exothermic DTA peaks. The degradation was preceded by a sharp endothermic peak at 123.3 °C, associated with melting metoprolol tartrate.

**Figure 4:**
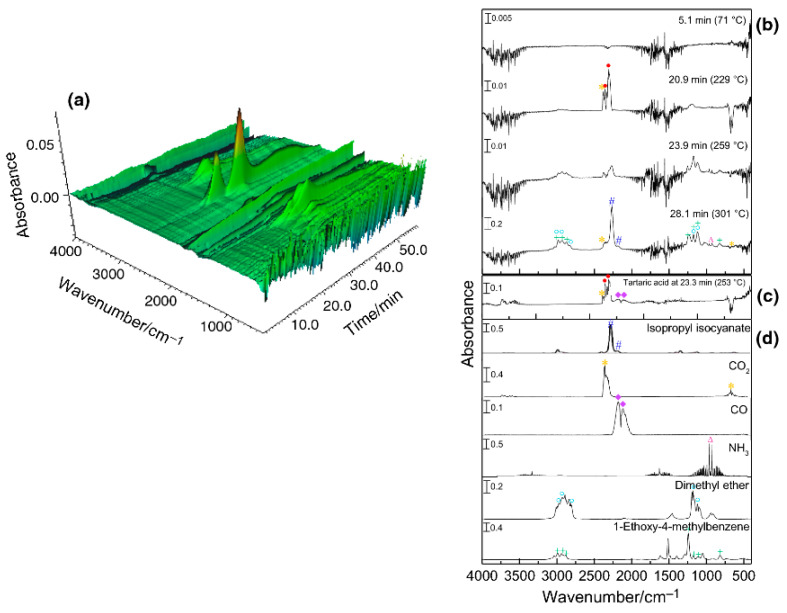
FTIR spectra of volatile products collected during thermal degradation of metoprolol tartrate using TG–FTIR system. (**a**)—three-dimensional spectrum of volatile degradation products as a function of time; and two-dimensional spectra: (**b**)—volatile degradation products collected at different times/temperatures; (**c**)—volatile degradation products of tartaric acid collected at 23.3 min/253 °C; and (**d**)—reference spectra of selected volatile degradation products (isopropyl isocyanate, carbon dioxide, carbon monoxide, ammonia, dimethyl ether and 1-ethoxy-4-methyl benzene) obtained from NIST and Nicolet TGA Vapor Phase databases. Reprinted with permission from Ref. [31], 2023, Springer.

The TG–FTIR coupled system was used to identify gaseous degradation products formed during thermal decomposition of metoprolol tartrate. The interpretation of the obtained data is shown in Figure 4. FTIR spectra of volatile degradation products were compared with reference spectra obtained from the NIST and Nicolet TGA Vapor Phase databases. Based on this methodology, TG–FTIR in nitrogen confirmed the presence of carbon monoxide, carbon dioxide and water in the first stage of mass loss. This indicates the degradation of tartaric acid. The theoretical mass loss associated with the degradation of this acid is consistent with the mass loss in the first stage of degradation. In the second stage of mass loss, dimethyl ether, 1-ethoxy-4-methylbenzene, isopropyl isocyanate, carbon dioxide and ammonia were identified. In addition, HPLC/MS analysis of the solid thermal decomposition products obtained by heating metoprolol tartrate to 290 °C suggests that intramolecular interaction in the liquid phase leads to the formation of molecules with higher mass than metoprolol. Combining all the results, it was found that the heated substance melts, followed by the decomposition of tartaric acid, the degradation of metoprolol with the formation of dimers and the destruction of the resulting structures with the formation of a coked residue that burns at high temperatures.

The mechanism of thermal degradation of tenofovir disoproxil fumarate (bis {[(isopropoxycarbonyl)-oxy]methyl} ({[(2*R*)-1-(6-amino-9*H*-purin-9-yl)-2propanyl]-oxy} methyl) phosphonate fumarate) was also investigated, using TG–FTIR to identify volatile degradation products and HPLC and LC/MS to study solid residues [22]. Endothermic DSC peaks indicated that the substance under study melts in nitrogen at 110.9 °C, while it melts in the air at 110.7 °C. Melting is followed by decomposition, and the second endothermic DSC peak indicates thermal destruction as the main degradation pathway.

TG studies have shown that the decomposition of tenofovir disoproxil fumarate is accompanied by three stages of mass loss. They occur in the temperature ranges 138–195 °C, 195–415 °C and 415-approx. 800 °C, with mass losses of, respectively: 35%, 25% and 23% in nitrogen and 34%, 23% and 23% in air. Stage one can be characterized as a rapid degradation process, while stage two reflects slow degradation. The degradation process in this stage is complex, as indicated by several small DTG peaks. Stage three, on the other hand, is a very slow process.

Comprehensive studies realized using TG–FTIR (identification of volatile degradation products), HPLC and LC/MS (identification of solid degradation products) and quantum chemistry methods (prediction of the chemical structure of degradation products) made it possible to develop the mechanism of destruction of tenofovir disoproxil fumarate. It was shown that in both atmospheres, the mechanism of degradation in the first two stages of mass loss is very similar. The first stage begins with the degradation of the ester bond, followed by the degradation of the phosphate disoproxil group. This is the main degradation process in this stage, which completes the beginning of fumaric acid decomposition, simultaneously starting stage two. The main process in this stage is the degradation of the tenofovir grouping and partially the adenine grouping. In turn, in stage three, the adenine ring is degraded with the formation of a coked residue.

### 3.2. TG Coupled to Mass Spectrometry

Another coupled thermal technique used to study pharmaceuticals is TG–MS. TG–MS is distinguished by the fact that it allows not only the identification, but also the analysis of the chemical structure of volatile degradation products based on mass spectra (*m*/*z* ratio), while TG–FTIR provides only the identification of degradation products based on characteristic absorption bands associated with the presence of specific functional groups in the molecule. Numerous examples of the application of TG–MS, among others, in the study of nanomaterials, nanocomposites, polymers and other materials are presented in review papers [14,49].

**Figure 5:**
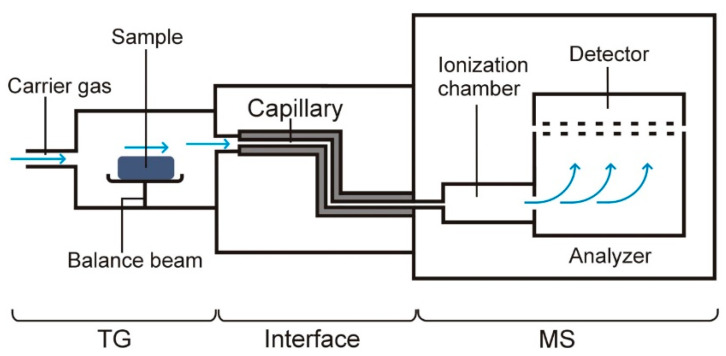
A block diagram of the equipment for TG–MS measurements.

A block diagram of the TG–FTIR equipment is shown in Figure 5. As with TG–FTIR, in addition to the TG and MS devices, the most important component of this system is the interface that connects the outlet of the TG oven to the inlet of the ionization chamber of the mass spectrometer (MS) [14,15,17]. The technical problem is that TG measurements are conducted at atmospheric pressure, while the MS has a high vacuum. Therefore, the connector must guarantee that the vacuum in the ionization chamber is maintained. The connector includes a capillary tube with the appropriate length and inner diameter. Through this capillary tube, volatile degradation products are transferred from the TG furnace to the MS ionization chamber, into which they are sucked by the pressure difference prevailing in the capillary tube and the ionization chamber. As in the case of TG–FTIR, the capillary tube is maintained at a certain temperature, which prevents condensation of volatile degradation products. The carrier gas is most often helium, but in some types of MS, it can also be air.

Based on the literature data, it can be concluded that coupled TG–MS systems are relatively rarely used to study pharmaceuticals. Only a few examples can be found, with the equipment used for exploring and the measurement conditions varying greatly. This is illustrated in Table 3.

The data compiled in Table 3 indicates that, as in the case of TG–FTIR, equipment from TA Instruments, model SDT 2960 DSC-TGA, and a mass spectrometer from Pfeiffer Vacuum, model Balzers ThermoStar Quadrupole Mass Spectrometry, were most commonly used for API studies. The authors of the cited publications, however, rarely provided detailed data on how the two instruments were combined and on MS analysis conditions. They did, however, provide data on TG analysis conditions. The most common sample masses used were 5–10 mg [42,47] and 15-20 mg [34]. In one case, the sample mass was as high as 161.5 mg [52]. Samples in open α-alumina sample holders [42,47] or platinum crucibles [34] were heated from ambient temperature to 300 °C [53], 400 °C [47,52], 500 °C [34] or 800 °C [42]; at heating rates of 5 °C/min [34,44,47], 10 °C/min [42,47,52,53] or 15 °C/min [47]; in helium [44], argon [47,52] or nitrogen [34,53]; and at flow rates of 16 mL/min [53], 20 mL/min [34,47], 100 mL/min [52] or 10 L/h [44].

The literature also describes a dual-coupled system that allows simultaneous analysis of TG, DSC, MS and FTIR in real time [57]. This system includes an apparatus for TG–DSC (STA 449 Jupiter, Netzsch), quadrupole mass spectrometer (OMS 403C Aëolos, Netzsch) and FTIR spectrometer (Tensor 27, Bruker). The TG–DSC device is connected to both spectrometers via a transfer line, through which volatile degradation products are simultaneously and continuously delivered to the QMS and FTIR devices. Both QMS and FTIR allow analysis of gaseous degradation products, with the difference that QMS provides qualitative and quantitative composition information, while FTIR allows identification of functional groups along with information on the aliphatic or aromatic nature of the gaseous products. By exploring natural polymers with complex chemical compositions isolated from soils that differ in chemical composition, the suitability of the TG–DSC–QMS–FTIR device for studying the chemical properties and thermal stability of fulvic and humic acids was tested.

Examples of the application of TG–MS in the study of pharmaceuticals are shown in Table 4. An interesting solution is the use of atmospheric pressure photoionization (APPI) for the analysis of acetylsalicylic acid tablets from different manufacturers [54]. The mass spectra obtained made it possible to detect the active ingredient in all tablets under study and to identify the tablet manufacturers. The data obtained showed that the developed new analytical technologies could be potentially useful in the quality control of pharmaceutical preparations and the identification of adulterated products.

In turn, the thermal decomposition of lornoxicam ((3*E*)-6-chloro-3-[hydroxy (pyridin-2-ylamino)methylene]-2-methyl-2,3-dihydro-4*H*-thieno [2,3-*e*] [1,2] thiazin-4-one 1,1-dioxide) can serve as an example for the analysis of volatile and solid degradation products formed both in inert nitrogen and an oxidizing air atmosphere [42]. Lornoxicam melts at 225–230 °C with simultaneous decomposition, which proceeds in two stages with significant mass losses. The first stage is accompanied by a 63–64% mass loss in the temperature range of 205–360 °C, regardless of the type of atmosphere (nitrogen, air). The mass loss in stage two is strongly influenced by the atmosphere. In nitrogen, there is slow pyrolysis in the temperature range of 360–800 °C, resulting in a small mass loss (15%). In the air, on the other hand, in a narrower temperature range (360–680 °C), oxidative degradation occurs, accompanied by a greater mass loss (39%). The exothermic DSC peak in this stage confirms the oxidation of the degradation products formed in stage one.

Volatile degradation products were analyzed using coupled DSC/TG–FTIR and TG–MS systems. Carbon dioxide, carbonyl sulfide, sulfur dioxide and hydrogen cyanide were found to be released in both air and nitrogen. In contrast, at higher temperatures, the qualitative and quantitative composition of volatile degradation products was strongly influenced by the atmosphere. Carbon dioxide and sulfur dioxide were detected in nitrogen, while carbon monoxide, carbon dioxide, nitrous oxide, carbonyl sulfide and sulfur dioxide were detected in air. Solid degradation products were also studied using LC/MS/MS. These were obtained by heating lornoxicam in a DSC/TG device under isothermal conditions at temperatures of 180, 220 and 235 °C. Three degradation products with complex chemical structures were identified from the mass spectra, two at 220°C—[M+H]^+^ *m*/*z* = 336, MS/MS 121 and [M+H]^+^ *m*/*z* = 356, MS/MS 148, 121, and the third at 235 °C—[M+H]^+^ *m*/*z* = 310, MS/MS 216, 95.

The effects of the chemical structure and geometric configuration of citric acid and aconitic acid isomers on the stability of decomposition products and the direction of thermal transformation were also studied using TG–FTIR and TG–MS coupled systems [47]. TG and DSC showed that cis-aconitic acid showed the lowest stability, while citric and trans-aconitic acids showed similar stability, much higher than the stability of cis-aconitic acid. This indicates that the configurational isomerism of aconitic acid has a significant effect on its thermal decomposition. The study also showed that citric and trans-aconitic acids can undergo thermal transformation (dehydration) directly to trans-aconitic anhydride. This transformation suggests that the geometrical configuration has a strong influence on the degradation pathway of aconitic acid isomers. Trans-aconitic anhydride, in turn, undergoes decarboxylation to itaconic anhydride, citraconic anhydride or a mixture of the two.

### 3.3. TG Coupled to GC/MS

TG–GC/MS is another coupled thermal technique classified in the group of non-continuous simultaneous techniques, i.e., it involves the studying of the same sample using two or more coupled measurement techniques when the collection of material to be explored for the other technique is not continuous. For this reason, measurements via TG–GC/MS do not take place in real time.

**Figure 6:**
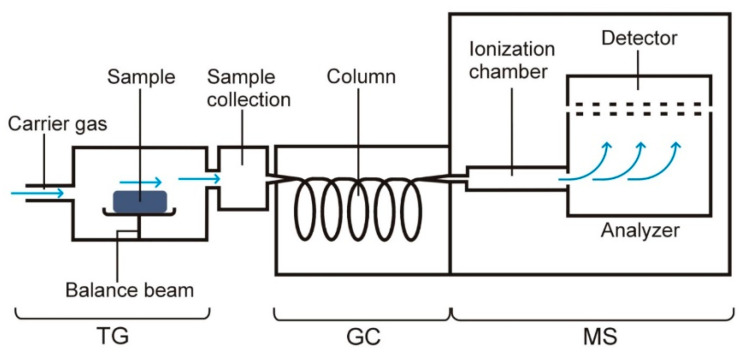
A block diagram of the equipment for TG–GC/MS measurements.

A block diagram of the equipment for TG–GC/MS is shown in Figure 6. Volatile degradation products that are released from the sample during heating are properly collected over specific temperature ranges in an appropriate manner and then fed into GC/MS [14,15]. The main advantage of the coupled TG–GC/MS system is that it provides information on the chemical structure of volatile degradation products, while TG–FTIR only allows the identification of volatile degradation products.

There are few examples in the literature of the application of coupled TG–GC/MS systems in the study of pharmaceuticals. One of them presents the results of zidovudine (azidothymidine, 1-[(2*R*,4*S*,5*S*)-4-azido-5-(hydroxymethyl)oxolan-2-yl]-5-methyl pyrimidine-2,4-dione) [58]. TG, DTG and DSC studies under a nitrogen atmosphere showed that the thermal decomposition of zidovudine occurs in three stages, for which mass losses and temperature ranges are as follows: 51.8%, 153–249 °C; 20.3%, 249–357 °C; and 28.2%, 360–650 °C. Degradation is preceded by the melting of the substance at 126.6 °C.

Volatile degradation products were obtained by heating zidovudine in the temperature range of 30–900 °C in a dynamic helium atmosphere. They were adsorbed onto Tenax 60/80 mesh porous polymer adsorbent and cooled to dry-ice temperature. After desorption at 300 °C, the volatiles were separated on a gas chromatograph coupled to a mass spectrometer under a helium atmosphere. Identifying the chemical structure of the volatile products, the mass spectra of the GC-evolved compounds were compared with standards from the National Institute of Standards (NIST) database. The presence of 2-furanomethanol and furan was found. In turn, using elemental analysis (C, H and N), IR, PXRD and DSC, it was suggested that the solid decomposition product of zidovudine in the first decomposition step is thymine. Such a mechanism confirms the presence of 2-furanomethanol among the volatile degradation products. In the second stage, thymine is degraded to form an intermediate product with fragments of the thymine structure. In the third stage, the coked residue burns.

Reliable analysis of volatile degradation products can also be performed using coupled systems consisting of a pyrolyzer and GC/MS (Pyr-GC/MS). An example is the study of the thermal degradation of simvastatin ((1*S*,3*R*,7*S*,8*S*,8a*R*)-8-{2-[(2*R*,4*R*)- 4-hydroxy-6-oxotetrahydro-2*H*-pyran-2-yl]ethyl}-3,7-dimethyl-1,2,3,7,8,8a-hexahydronaphthalen-1-yl 2,2-dimethylbutanoate) [59]. Successive samples corresponding to single crystals of simvastatin were placed in a platinum crucible and pyrolyzed under isothermal conditions at temperatures of 200, 250, 300, 400 and 550 °C. Volatile products obtained at the specified temperature were introduced into GC/MS using helium as a carrier gas. Fragmentation was carried out with electron ionization, and the mass spectrometer was operated in SCAN mode, scanning the *m*/*z* range of 50–550. Data obtained from Pyr-GC/MS analysis indicated a good correlation between pyrolysis mass losses and thermal decomposition by TG. The identification of new compounds based on the signals in the mass spectra (peaks at *m*/*z* 284 and 207) formed during the first stage of decomposition indicates the complexity of the degradation process and explains the difficulty in determining the sequence of reactions during the isothermal decomposition of simvastatin.

Coupled Pyr-GC/MS systems were further used to study the thermal degradation processes of tacrolimus [60], fluconazole [61], efavirenz [62] and raw medicinal plant material [63]. However, it should be noted that pyrolysis is not a thermal analysis technique. In this method, no physical properties of the samples are recorded, and thermal decomposition does not take place under conditions of programmed temperature changes. Therefore, only a brief description of this technique is given, without further discussion of the use of Pyr-GC/MS in the study of pharmaceuticals.

## 4. Simultaneous DSC Measurements

As already mentioned, reliable interpretation of DSC curves is often difficult, including because DSC is a non-specific technique and because of overlapping peaks. Therefore, new design solutions combining thermal analysis techniques with non-thermal techniques have been introduced. They made it possible to directly observe the samples during heating, monitor changes in the chemical composition of the samples and identify their crystalline state, thus facilitating the interpretation of DSC curves.

### 4.1. DSC–Photovisual

**Figure 7:**
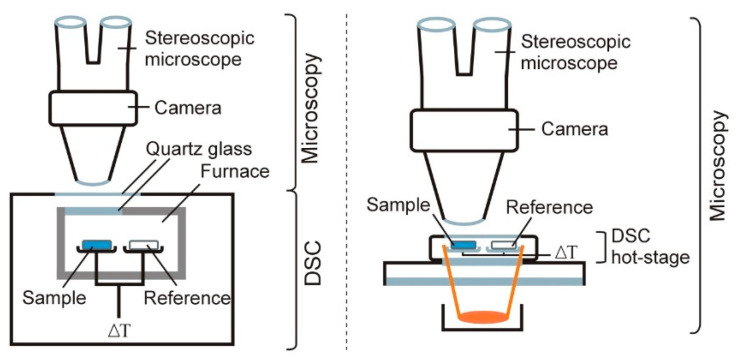
A block diagram of the equipment that allows observation of the sample during DSC analysis.

One of the first solutions that allowed direct observation of samples during heating was the combination of an optical microscope with a heating table, which provided heating of the explored samples under controlled conditions. This is now the well-known Hot-Stage Microscopy (HSM) technique, with an established position in the field of thermal research. Details of the design of HSM instruments and an exhaustive characterization of the application of HSM in the study of pharmaceuticals are presented in several review papers [64,65,66,67]. Since the weakness of HSM is the inability to simultaneously record the DSC curve together with the observation of changes in the sample during heating, devices have been constructed that allow the simultaneous performance of both functions. Of these solutions, DSC–photovisual is the most widely used and, at the same time, is an extremely useful tool for studying pharmaceuticals. A block diagram of this device is shown in Figure 7.

In the simplest case, a window made of quartz glass is placed in the lid of the DSC oven, which allows direct observation of the thermal transformations occurring in the sample during DSC analysis. The quartz window can be connected to a charge-coupled device (CCD) digital camera. The CCD camera can also be connected to the window for DSC through a stereoscopic microscope, i.e., an optical microscope with separate eyepieces that make the magnified image seen in them three-dimensional. The magnification range of stereoscopic microscopes varies from 3× to more than 200×.

The above-described solution found its way into commercially available devices, such as a DSC-50 photovisual system (Shimadzu) [18,68,69,70,71,72,73,74,75,76,77,78] and TG-DSC 1 (Mettler-Toledo) [37,39,40,42]. The literature also describes a commercially available TG/DTA device (Seiko) that allows three simultaneous measurements: TG, DTA and observation of the sample during heating [79]. The sample is observed with light microscopy through a double quartz layer window to permit a clear view into the furnace of the TG/DTA device.

In another solution, a heating table is placed on the table of the optical microscope to allow the heating of the sample in a controlled way (HSM). Mettler has developed two types of heating tables, HS82 (Microscope hot-stage) and HS84 (DSC hot-stage) [80]. Both tables allow heating of the sample to 375 °C. However, only the HS84 heating table is equipped with the corresponding FRS 5 DSC ceramic sensors, and its operation is controlled by the FP 80HT central digital temperature programmer, which allows DSC measurements. The HS84 heating table thus fulfills the role of a miniaturized device for DSC.

A detailed description of the equipment and conditions for the study of pharmaceuticals using the DSC–photovisual method can be found in Table 5. The data presented indicate the high utility of both devices, i.e., DSC-50 photovisual system (Shimadzu) and TG-DSC 1 (Mettler-Toledo), coupled with a digital camera and/or microscope for the study of APIs, excipients and solid dosage forms, especially tablets. Most studies were performed at temperatures up to approx.. 300°C under a nitrogen atmosphere. Most commonly used were 2–3 mg samples placed in an open α-alumina crucible [37] or aluminum pan [39,59].

The use of simultaneous DSC–photovisual studies facilitates the identification of phase transitions, especially those involving the solid phase [64,65,66,67]. Characterizing these transitions with DSC alone would be very difficult or impossible. DSC–photovisual makes it possible to observe phase transitions such as melting, evaporation, sublimation, crystallization, glass transition, polymorphic and crystalline-amorphous transitions, and also enables characterization of thermal processes such as dehydration and desolvation, physical and chemical interactions between ingredients and thermal degradation and decomposition.

Table 6 summarizes selected examples of the application of DSC–photovisual measurements to the study of pharmaceuticals. They indicate a wide range of research problems that visual observation of a sample during DSC analysis can be useful for solving. Predominant studies of the behavior of samples during heating at lower temperatures can be particularly useful for identifying phase transitions, detecting interactions between ingredients of pharmaceutical preparations and thermal degradation and thermal stability.

Thiabendazole (4-(1*H*-1,3-benzodiazol-2-yl)-1,3-thiazoles) is a good example of using DSC–photovisual to study the thermal stability of APIs [73]. DSC showed that thiabendazole melts at 304 °C, while TG indicated that thiabendazole degrades in an air atmosphere, with complete mass loss in the temperature range 201–436 °C. This implies that thiabendazole melting is preceded by a 30–40% mass loss, which can be linked to the disengagement of the thiazolyl group from the thiabendazole structure. Therefore, the endothermic DSC peak at 304 °C does not reflect the melting of thiabendazole, but the melting of the newly formed product, whose structure has not been determined. The DSC–photovisual system highlighted the color change of the sample at 240 °C, confirming the degradation of thiabendazole. Thus, DSC–photovisual perfectly illustrated the changes occurring in thiabendazole during heating.

### 4.2. DSC–FTIR Microspectroscopy and DSC–XRD

The DSC can also be coupled to an FTIR microspectrometer, enabling the recording of DSC curves and FTIR spectra in a single experiment. In terms of design, the DSC–FTIR equipment consists of an FTIR microspectrometer coupled to an optical, transmission or reflection microscope. The DSC device (HS84 DSC hot-stage) is placed on the microscope table.

A detailed description of the equipment and conditions for exploring pharmaceuticals using DSC–FTIR microspectroscopy is presented in Table 5. Attention should be paid to the method of sample preparation for DSC–FTIR, which differs significantly from the procedures used in conventional DSC. The method described in the literature is that a trace of the sample powder was either smeared on the surface of one piece (or directly pressed to one piece) of KBr pellets without further compression or sealed into two pieces of KBr pellets and then directly compressed with an IR spectrophotometric hydraulic press [82,85,91,92]. The former procedure is known as the smeared method or 1 KBr method, and the latter one is known as the compressed method or 2 KBr method. The KBr pellets were compressed for 15 s to form a disc with a compression pressure of 200 kg/cm^2^ [82,85] or 400 kg/cm^2^ [91,92].

Selected examples of the application of DSC–FTIR microspectroscopy in pharmaceutics are summarized in Table 6. Analysis of these data indicates that simultaneous recording of FTIR spectra and DSC curves makes it possible to identify the formation of intra- or intermolecular hydrogen bonding or changes in these bonds during heating. Changes in FTIR spectra in conjunction with endo or exothermic DSC peaks make it possible to infer the nature of polymorphic transitions, distinguish sublimation from degradation, study intramolecular cyclization processes associated with lactam or anhydride formation and quickly identify co-crystals that form in physical mixtures of API and coformer during heating. A comprehensive discussion of examples of the application of DSC–FTIR microspectroscopy for rapid examination of active substances’ stability in the solid state [95], screening and detection of pharmaceutical co-crystals [96] and potential application for pharmaceutical analysis [97] is presented in the review papers.

**Figure 8:**
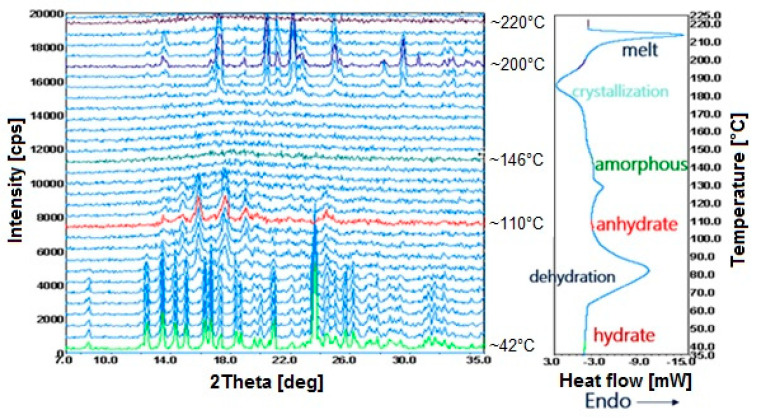
Simultaneous DSC–XRD measurement for a selected organic drug substance [98]. Changes in X-ray diffractograms and DSC curve due to dehydration, amorphization, crystallization and melting.

For simultaneous DSC–XRD measurements, equipment from Rigaku is available, which consists of an X-ray powder diffractometer integrated with a DSC device [98]. As shown in Figure 8, simultaneous DSC–XRD measurements performed in a single experiment showed that phase transitions such as dehydration, amorphization, crystallization and melting were taking place in the sample. Using the example of the phase and polymorphic transitions of ganciclovir (2-amino-9-(1,3-dihydroxypropan-2-ylxymethyl) -3*H*-purin-6-one), it was also shown that simultaneous DSC–XRD measurements are essential to better understand these transitions [94]. This method allows detailed characterization of the structural changes occurring in the samples as an effect of temperature.

Highly specialized DSC–XRD studies have also been carried out using a synchrotron X-ray source [99]. The synchrotron’s high-energy X-rays allow the diffractogram to be recorded in less than 2 s. As a result, accurate quantitative data on thermally induced phase transitions and additional information on the nature of phase transitions can be obtained. Current XRD instruments do not provide such detailed data, as they require a much longer time to record the diffractogram. The utility of the new technique was demonstrated by exploring two model systems consisting of glutaric acid and sulfathiazole; both drug substances exhibit enantiotropic polymorphism. It was shown that the phase transitions occurring between the low-temperature and high-temperature polymorphic forms of the samples are direct solid–solid transformations. The resulting diffractograms also make it possible to determine the composition of phase fractions and unit cell parameters as a function of temperature. Thus, the combination of DSC with synchrotron XRD made it possible to identify the phase composition of the test sample, which was a mixture of phases and not, as it seemed on the basis of DSC measurements, a pure phase.

## 5. Conclusions

The results obtained with coupled TG–FTIR, TG–MS and TG–GC/MS systems are crucial for drug formulation technology. They make it possible to know exactly how pharmaceutical substances behave during heating, identify volatile degradation products and determine the mechanism of thermal degradation. These data make it possible to predict the behavior of APIs during the manufacture of drug formulations and make it possible to determine their shelf life and storage conditions. On the other hand, simultaneous DSC–photovisual, DSC–FTIR microspectroscopy and DSC–XRD measurements allow direct observation of the samples during heating, monitoring changes in the chemical composition of the samples or identifying their crystalline state, thus facilitating the interpretation of DSC curves. This is of particular importance since DSC is a non-specific technique.

## Figures and Tables

**Table 2:** Selected examples illustrating the application of TG–FTIR coupled systems and supporting techniques in the study of active pharmaceutical ingredients.

Active Pharmaceutical Ingredients	Therapeutic Activity	Purpose of Research	Supporting Techniques	References
Atenolol	antihypertensive drug	volatile degradation products	TG/DSC	[24]
Sulindac acid	nonsteroidal anti-inflammatory agent	thermal behavior, volatile degradation products, thermal stability	TG/DSC, DSC–photovisual, FTIR, NIR, UV-Vis	[37]
Amlodipine besylate	antihypertensive drug		TG/DSC, DSC	[25]
Amiodarone hydrochloride	antiarrhythmic and vasodilatory drug		TG, DSC, Flash DSC, XRPD	[43]
Di-disubstituted 1,2,4-triazoles	potential antitumor and antibacterial drug		TG/DTG/DSC	[46]
Cephalosporins	antibacterial drug	thermal decomposition, volatile degradation products, the kinetics of thermal decomposition	TG/DTG/DTA, DSC	[45]
Enoxacin, hydrochloride			TG/DTG/DSC, FTIR	[26]
Nicotinic acid	B group vitamin			[27]
Carbamazepine	antiepileptic drug	impact of sample pans and atmosphere on the kinetics of thermal decomposition	TG/DTG/DTA, DSC, FTIR, HPLC	[33]
Naproxen, ketoprofen	nonsteroidal anti-inflammatory agents	thermal decomposition, volatile degradation products, mechanism of thermal decomposition	TG/DTG/DTA, DSC, HSM	[29]
Oxytetracycline hydrochloride	antibiotic		TG/DTG/DTA, DSC	[30]
Carvedilol	antihypertensive drug			[28]
Atenolol, nadolol				[36]
Metoprolol tartrate	antihypertensive drug	thermal decomposition, volatile and solid degradation products, mechanism of thermal decomposition	TG/DTG/DTA, DSC, HSM, HPLC-MS	[31]
Labetalol			TG/DTG/DTA, DSC, HSM, GC/MS	[32]
Tenofovir disoproxil fumarate	antiretroviral agent		TG/DTG/DSC, FTIR, HPLC, LC-MS	[22]
Famciclovir				[23]
Capecitabine	antitumor drug			[25]
Epinephrine	neurotransmitter		TG/DTG/DTA, DSC, MS, PXRD, FTIR	[38]
Tryptophan methyl ester	amino acid ester, the precursor of bioactive compounds		TG/DTG/DTA, TG/DSC, FTIR, NMR, HPLC-MS	[41]
Norfloxacin, co-crystal with saccharin, solvate	antibiotic	impact of solvent and synthesis method on the co-crystallization product	TG/DTA, DSC, DSC–photovisual, PXRD, FTIR	[39]
Meloxicam, co-crystals with organic acids	nonsteroidal anti-inflammatory agent	thermal behavior, identification of co-crystal composition, polymorphic transitions	TG/DSC, DSC–photovisual, PXRD, FTIR, Raman	[40]

**Table 3:** TG–MS coupled systems used in the study of active pharmaceutical ingredients.

TG Instruments	Mass Spectrometers	Transfer Line, MS Measurements	References
SDT 2960 DSC-TGA (TA Instruments)	Balzers ThermoStar GSD 300T Quadrupole MS (Pfeiffer Vacuum)	heated silica capillary; SAC mode, MID mode	[44,50,51]
STA 409 Thermobalance (Netzsch)	Leybold Infiction 200 MS	capillary coupling; 150 °C	[52]
STA 449 Jupiter F3 TGA-DSC/DTA (Netzsch)	QMS 403 C Aëolos Quadrupole MS (Netzsch)		[47]
TGA/SDTA 1150 (Mettler-Toledo)	HPR20 Quadrupole MS (Hiden)	capillary coupling; 250 °C; nitrogen, air, flow rate 60 mL/min; EI, 70 eV, SEM	[42]
Setsys 16/18 Evolution TG-DTA/DSC (Setaram)	Balzers ThermoStar Quadrupole MS (Pfeiffer Vacuum)	capillary coupling; 198 °C	[34]
Setsys 16/18 Evolution TG-DTA/DSC (Setaram)	ThermoStar GSD 301T Quadrupole MS (Pfeiffer Vacuum)		[53]
STA 7200 TG/DTG/DTA (Hitachi)	Chromaster 5610 MS Detector (Hitachi)	l = 500 mm, highly flexible, small dead volume, heated via electric resistance; APPI	[54,55]
Thermo plus EV02 TG-DTA (Rigaku)	Photo ionization Mass Spectrometer (Rigaku)	quartz capillary; 200 °C; helium, flow rate 100 mL/min; PI; 70 eV; m/z range 1–400	[56]

APPI—atmospheric pressure photo ionization; EI—electron ionization; l—length of transfer line; MID—multiple ion detection mode; PI—photo ionization; SAC—scan analog mode; SEM—secondary emission multiplier detector; 70 eV—kinetic energy of ionized electrons.

**Table 4:** Selected examples illustrating the application of TG–MS coupled systems and supporting techniques in the study of active pharmaceutical ingredients.

Materials Examined	Therapeutic Activity	Purpose of Research	Supporting Techniques	References
Human hair	potential diagnostic material	preliminary thermal characteristics of various hair samples	TG/DTA, DSC	[50]
Biofilms of the strain of *Pseudomonas aeruginosa*	potential diagnostic material	identification of the Pseudomonas aeruginosa strain under clinical conditions	TG/DTG/DTA	[55]
Acetylsalicylic acid, drug formulations	nonsteroidal anti-inflammatory agent	quality control and identification of falsified drug formulations	TG/DTG/DTA	[54]
Cyclodextrins, natural and modified	excipients	thermal behavior, the difference in fragmentation profiles of native and substituted products	TG/DTA, XRPD	[51]
Ciprofloxacin, salts	antibacterial drugs	thermal behavior, rearrangement of hydrogen bonds	TG, DSC, FTIR, EA	[52]
Lactic acid	used in skin, gastrointestinal and gynecological diseases	thermal decomposition, kinetics of thermal decomposition	TG/DTG, DSC	[53]
Torasemide	diuretic and antihypertensive drug	crystal transitions, solvate, volatile degradation products, mechanism of thermal decomposition	TG/DTG, DTA, TG–FTIR, HSM, SEM	[44]
Salbutamol sulfate	used in acute and chronic bronchoconstriction	thermal decomposition, volatile degradation products, mechanism of thermal decomposition	TG/DTG, DSC, TG–FTIR	[34]
Lornoxicam	nonsteroidal anti-inflammatory agent	thermal decomposition, volatile and solid degradation products, mechanism of thermal decomposition	TG/DSC, TG–FTIR, DSC–photovisual, HSM, PXRD, LC-MS/MS	[42]
Citric acid, isomers of aconitic acid	excipient	impact of chemical structure and geometrical configuration of acids on the degradation products’ stability and the course of their decomposition	TG, TG–FTIR, DSC	[47]
1-Hydroxy-7-azabenzotriazole	peptide coupling reagent	thermal decomposition, volatile and solid degradation products, mechanism of thermal decomposition, thermal safety during manufacture, transport, use and storage	TG, DSC, ARC, FTIR, SEM-EDS, XPS	[56]

ARC—accelerating rate calorimeter; EA—elemental analysis; SEM-EDS—scanning electron microscopy-energy dispersive spectra; XPS—X-ray photo electron spectroscopy.

**Table 5:** DSC–photovisual, DSC–FTIR microspectroscopy and DSC–XRD simultaneous systems used in the study of pharmaceuticals.

DSC Instruments	Second Techniques	Measurement Conditions	References
**DSC–photovisual**			
DSC-50 photovisual system (Shimadzu)	microscope (Olympus), microscope SZ-CTV60 (Olympus); camera VCC-D520 (Sanyo), camera VCC-520 (Sony)	β = 5 °C/min, 10 °C/min, 15 °C/min, 20 °C/min; ΔT = 20–250 °C, 25–300 °C, 25–400 °C, 25–500 °C; nitrogen, flow rate 50 mL/min	[18,68,69,70,71,72,73,74,75,76,77,78]
TG-DSC 1 (Mettler-Toledo)	camera SC30, 3.3-megapixel CMOS sensor, 6.5 × zoom (Olympus)	β = 10 °C/min; 20–225°, 20–300 °C; nitrogen, air, flow rate 50 mL/min	[37,39,40,42]
**DSC–FTIR microspectroscopy**			
HS84 DSC hot-stage system (Mettler)	FTIR microscopic spectrometer Micro FTIR-200 (Jasco)	β = 3 °C/min, 5 °C/min; ΔT = 25–160 °C, 25–270 °C, 30–120 °C, 30–200 °C, 30–300 °C; transmission mode; MCT detector	[19,81,82,83,84,85,86,87,88]
HS84 DSC hot-stage system (Mettler)	FTIR microscopic spectrometer IRT-5000-16/FTIR 6200 (Jasco)	β = 3 °C/min; ΔT = 30–200 °C, 30–240 °C, 30–250 °C, 30–300 °C, 30–320 °C; transmission mode; MCT detector, 4 cm^–1^ resolution	[89,90,91,92,93]
**DSC–XRD**			
Thermo Plus DSC (Rigaku)	SmartLab Multipurpose Diffractometer (Rigaku)	β = 5 °C/min; ΔT = 25–350 °C; nitrogen, flow rate 50 mL/min; X-ray source Cu K_α_; step size 0.02° between 3 and 35° (2θ)	[94]

MCT—mercury cadmium telluride detector; β—heating rate; ΔT—temperature range of sample examination.

**Table 6:** Selected examples illustrating the application of DSC–photovisual, DSC–FTIR microspectroscopy and DSC–XRD simultaneous systems and supporting techniques in the study of pharmaceuticals.

Pharmaceuticals	Therapeutic Activity	Purpose of Research	Supporting Tools	References
**DSC–photovisual**				
Powdered starch	excipient	evaluation of the gelatinization process	DSC	[68]
Indinavir sulfate	antiretroviral agent	thermal behavior, quality assessment	DSC, TG/DTG	[78]
Prednisone, tablets	anti-inflammatory and immunosuppressant agent	thermal behavior, interactions between tablets’ ingredients	DTA, DSC, TG, XRPD, FTIR	[69]
Dipyrone sodium, caffeine, orphenadrine citrate	analgesics drugs			[18]
Hydrochlorothiazide, tablets	diuretic used in hypertension	thermal behavior, interactions between tablets’ ingredients, thermal stability, the kinetic parameters	DSC, TG	[70]
Propranolol hydrochloride, tablets	used to treat heart problems			[71]
Cimetidine, tablets	used in peptic ulcer disease and indigestion			[75]
Metronidazole, tablets	antibiotic (gastrointestinal infections)			[76,77]
Thiabendazole, tablets	antifungal and antiparasitic agent		DSC, TG, FTIR	[73]
Quercetin, rutin	health-promoting flavonoids	thermal decomposition, the kinetic parameters	DSC, TG	[72]
Simvastatin	used in hypercholesterolemia	thermal decomposition, volatile degradation products, thermal stability, the kinetic parameters	DSC, TG, Pyr-GC/MS	[59]
Fluconazole	antifungal drug			[61]
Efavirenz	antiretroviral agent			[62]
Crude extract of *Albizia inopinata*	potential antihypertensive and vasodilation action	influence of stabilizers on the thermal decomposition, kinetic parameters	DSC, TG	[74]
**DSC–FTIR microspectroscopy**				
Acetaminophen	analgesic and antipyretic drug	effect of temperature on the intermolecular hydrogen bonding at solid and liquid states	DSC	[19]
Famotidine	histamine H_2_-receptor antagonist	effect of grinding on the polymorphic transitions	DSC, FTIR	[83]
Gabapentin	anticonvulsant agent	heat-induced polymorphic interconversions	DSC, TG, PXRD, FTIR	[86]
		heat-induced intramolecular lactamization, kinetics	DSC, TG, FTIR	[85]
Enalapril maleate	used in hypertension and congestive heart failure	formation of diketopiperazine via heat-induced intramolecular cyclization	DSC, TG	[81]
Eudragit E	excipient	heat-induced intramolecular anhydride formation		[89]
Nitroxoline	urinary antibacterial drug	solid-state characteristics, sublimation, kinetic parameters	DSC, TG, FTIR	[87]
Metoclopramide hydrochloride	used in stomach and esophageal problems	thermal behavior (dehydration, amorphization, recrystallization)	DSC, TG	[90]
Trehalose dihydrate	excipient	dehydration, rehydration, polymorphic transition	DSC, TG	[82]
10-Hydroxycamptothecin	used in cancer therapy	thermal behavior (dehydration, rehydration, decarboxylation, polymorphism), thermal stability	DSC, TG, FTIR, ES-IT-MS	[84]
Co-crystals of APIs with various co-formers	improved physicochemical properties of APIs	direct screening of thermally-induced co-crystals’ formation via specific intermolecular interaction	DSC	[88,91,92,93]
**DSC–XRD**				
Ganciclovir	antiviral drug	effect of temperature on the structural changes of crystal forms, polymorphic transitions	DSC, TG/DTG, HSM, SEM, PXRD, FTIR, EA	[94]

EA—elemental analysis; ES-IT-MS—electron spray ion trap mass spectrometry; SEM—scanning electron microscopy.

## Data Availability

This is review article. In this article there are no research data generated by authors.
